# Human IDO2 exhibits unique binding affinities distinct to those of human IDO1

**DOI:** 10.1111/febs.70476

**Published:** 2026-03-10

**Authors:** Shunsuke Nogi, Ayumu Takahashi, So Murakami, Nami Adachi, Tina Fujimoto, Yohta Fukuda, Taku Yamashita, Tsuyoshi Inoue, Hirofumi Tsujino

**Affiliations:** ^1^ Graduate School of Pharmaceutical Sciences The University of Osaka Suita Japan; ^2^ Integrated Frontier Research for Medical Science Division, Institute for Open and Transdisciplinary Research Initiatives (OTRI) The University of Osaka Suita Japan; ^3^ Department of Pharmacy Mukogawa Women's University Nishinomiya Japan; ^4^ Center for Infectious Disease Education and Research (CiDER) The University of Osaka Suita Japan; ^5^ Museum Links The University of Osaka Toyonaka Japan

**Keywords:** crystal structure, heme protein, indoleamine 2,3‐dioxygenase, ligand‐binding position, spectroscopic methods

## Abstract

Indoleamine 2,3‐dioxygenase 2 (IDO2) is a heme enzyme in the kynurenine pathway that shares high structural similarity with IDO1 but exhibits markedly lower catalytic activity. To clarify the molecular basis of this difference, we performed spectroscopic, biochemical, and crystallographic analyses of human IDO2. We found that IDO2 binds L‐tryptophan (L‐Trp) in a flipped orientation stabilized by the IDO2‐specific residue His143, which results in inefficient catalysis. Replacement of His143 with tyrosine, the corresponding residue in IDO1, restored an IDO1‐like binding mode of L‐Trp and enhanced activity by more than 1000‐fold. Structural analyses further revealed that IDO2 accommodates various tryptophan derivatives, such as 5‐methyl‐l‐Trp (5MT) and 5‐methoxy‐l‐Trp (5MoT), in a productive conformation, while other ligands, including D‐Trp and serotonin, adopt nonproductive poses. In addition, we observed that 5MT and 5MoT are metabolized by IDO2 at levels comparable to the metabolism of L‐Trp by human tryptophan 2,3‐dioxygenase. These results highlight the unique structural constraints that underlie IDO2's low activity and broadened substrate recognition, providing a molecular framework for understanding the functional divergence between IDO1 and IDO2.

## Graphical abstract

Although indoleamine 2,3‐dioxygenase 2 (IDO2) is highly homologous to IDO1, it displays markedly lower catalytic activity. We found that IDO2 binds L‐tryptophan (L‐Trp) in a flipped orientation stabilized by the IDO2‐specific residue His143. Replacement of His143 with the IDO1‐equivalent tyrosine restored an IDO1‐like binding mode and increased catalytic activity by more than 1000‐fold. IDO2 also binds 5‐methyl‐l‐tryptophan (5MT) productively and metabolizes it at a rate comparable to human tryptophan 2,3‐dioxygenase.

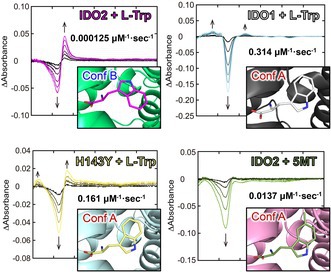

**Abbreviations**
3IPA: 3‐indolepropionic acid
5HT: serotonin (5‐hydroxytryptamine)
5HTP: 5‐hydroxy‐tryptophan
5MoT: 5‐methoxy‐l‐tryptophan
5MT: 5‐methyl‐l‐tryptophan
AhR: aryl hydrocarbon receptor
CO: carbon monoxide
D,L‐5MoT: 5‐methoxy‐d,l‐tryptophan
D,L‐5MT: 5‐methyl‐d,l‐tryptophan
D‐Trp: D‐tryptophan
IDO1: indoleamine 2,3‐dioxygenase 1
IDO2: indoleamine 2,3‐dioxygenase 2
IPTG: isopropyl β‐d‐1‐thiogalactopyranoside
L‐1MT: 1‐methyl‐l‐tryptophan
L‐5MoT: 5‐methoxy‐l‐tryptophan
L‐5MT: 5‐methyl‐l‐tryptophan
L‐Trp: L‐tryptophan
MBP: maltose‐binding protein
MBP‐IDO2: IDO2 containing an N‐terminal maltose‐binding protein tag
NFK: N′‐formylkynurenine
NMDA: N‐methyl‐d‐aspartate
NSCLC: nonsmall cell lung cancer
TDO: tryptophan 2,3‐dioxygenase
TRY: tryptamine
UV–Vis: ultraviolet–visible
WT: wild‐type

## Introduction

L‐tryptophan (L‐Trp), an essential amino acid, has three fates after absorption into the body. It is used for protein synthesis [1], utilized in the serotonin pathway for the synthesis of neurotransmitters [2], and metabolized through the kynurenine pathway. It is known that 95% of dietary L‐Trp is consumed via the kynurenine pathway [3]. The final products of this pathway include acetylcoenzyme A and nicotinamide adenine dinucleotides, which are indispensable for human survival [4].

Beyond its role in basic metabolism, recent studies have highlighted the association between intermediates of the kynurenine pathway and immune‐related and neurological disorders [5]. Kynurenine accumulation in immune cells induces immune tolerance by promoting the proliferation of regulatory T cells via the aryl hydrocarbon receptor (AhR) [6], which may also be activated by downstream metabolites of the kynurenine pathway, such as kynurenic acid [7]. Local depletion of L‐Trp has been suggested to induce immune tolerance by suppressing T helper 17 cell maturation via general control of the nonderepressible 2 kinase pathway [8].

The kynurenine pathway is also closely linked to neurological function. For example, kynurenic acid inhibits N‐methyl‐d‐aspartate (NMDA)‐type glutamate receptors present in the striatum and hippocampus [9], whereas quinolinic acid activates NMDA receptors [10], thereby influencing neural activity and contributing to the pathophysiology of psychiatric diseases.

Humans possess three L‐Trp oxygenases that regulate the rate‐limiting step of the kynurenine pathway: tryptophan 2,3‐dioxygenase (TDO), indoleamine 2,3‐dioxygenase 1 (IDO1), and indoleamine 2,3‐dioxygenase 2 (IDO2) [11]. All of these enzymes contain a heme cofactor at their active sites to bind to dioxygen. The dioxygen molecule bound to the heme is subsequently reduced to a superoxide species (O_2_^−^), which facilitates cleavage of the C2–C3 bond in the indole ring of L‐Trp, leading to the formation of N′‐formylkynurenine (NFK). Although they catalyze the same chemical reaction, their expression profiles and physiological functions differ.

TDO is expressed in the liver and contributes to systemic L‐Trp homeostasis via dietary L‐Trp metabolism [12]. It is also involved in brain L‐Trp metabolism, and mutations in exons or introns of the *TDO* gene have been linked to symptoms of neurological disorders, such as Tourette syndrome and attention deficit hyperactivity disorder [13].

IDO1 exhibits broader substrate specificity than TDO, metabolizing L‐Trp and other indole‐ring‐containing compounds [14]. IDO1 is broadly expressed in various tissues and immune‐related cells, including dendritic cells, macrophages, placenta, lung, and intestinal epithelium [15]. Its expression is induced by inflammatory cytokines such as Interferon‐γ [16], tumor necrosis factor‐α [17], and lipopolysaccharides [18]. Through these mechanisms, IDO1 plays a pivotal role in peripheral immune tolerance, tumor immune evasion, and chronic inflammation. Owing to these characteristics, IDO1 is a target molecule for cancer therapy, and extensive drug discovery research has been conducted. However, in a Phase III clinical trial conducted in 2018 to evaluate the combination therapy of the IDO1 inhibitor epacadostat and the PD‐1 inhibitor pembrolizumab, no significant difference in efficacy was observed compared to the pembrolizumab monotherapy group, leading to a lack of approval [19]. Therefore, targeting IDO1 alone is probably insufficient, highlighting the need to target other molecules [20].

IDO2, discovered in 2007 [21], shares 43% sequence identity with IDO1, but displays distinct expression and functional profiles. IDO2 is constitutively expressed in several tissues, including the liver, brain, kidneys, and gonads [22, 23], and its expression is upregulated by activation of AhR [24]. IDO2 is also known to be upregulated in several cancer tissues [25, 26, 27, 28]; however, the relationship between IDO2 and cancers is complicated. For example, studies in mouse models have shown that inhibition of the *Ido2* gene is protective in pancreatic ductal adenocarcinoma [29], but specific IDO2 genetic variants in humans are more frequent in nonsmall cell lung cancer (NSCLC) patients compared with controls and IDO2 expression is elevated in NSCLC tissues [30]. In addition, its role in immunity remains unclear. Some studies have shown a protective effect, whereas others have indicated worsening symptoms [31, 32]. IDO2 has also been implicated in neurological disorders, as *Ido2* knockout mice exhibit autism spectrum disorder‐like behaviors, suggesting its role in maintaining the homeostasis of L‐Trp metabolism and dopaminergic neurons [33]. Beyond its catalytic role, recent studies suggest that IDO2 acts as a signaling or regulatory protein. For example, IDO2 has been reported to modulate B‐cell function and autoantibody production through nonenzymatic mechanisms [34, 35].

IDO2, which has distinct roles from those of IDO1, holds potential as a useful drug target. Therefore, recent drug discovery research has focused not only on IDO1 but also on IDO2, with efforts directed toward designing both IDO2‐selective inhibitors [36] and dual inhibitors that simultaneously block IDO1 and IDO2 [37]. However, IDO2 exhibits extremely low L‐Trp metabolic activity, leaving its *in vivo* role unclear. Although two IDO2‐specific residues at the catalytic site, H143 and T184, are reported to be key residues for low activity [38], the detailed molecular reason remains unresolved because of the lack of atomic‐level structural information.

Taken together, despite increasing interest in IDO2, its physiological function remains incompletely understood, and several, sometimes conflicting, hypotheses have been proposed. These include the idea that IDO2 functions as an enzyme responsible for L‐Trp metabolism despite its low activity, or that its actual substrate differs from L‐Trp [39]. Other hypotheses suggest that IDO2 functions as a pseudoenzyme that controls IDO1 activity by isolating L‐Trp from IDO1 [30], or that it regulates IDO1 or other proteins nonenzymatically through protein–protein interactions [40]. The difficulty in deciphering the function of IDO2 and developing its inhibitors partially derives from the lack of experimental structures. In the present study, we aimed to elucidate the function of IDO2 by gathering detailed enzymatic and structural information.

## Results

### Spectroscopic and enzymatic properties of IDO2

**Fig. 1:**
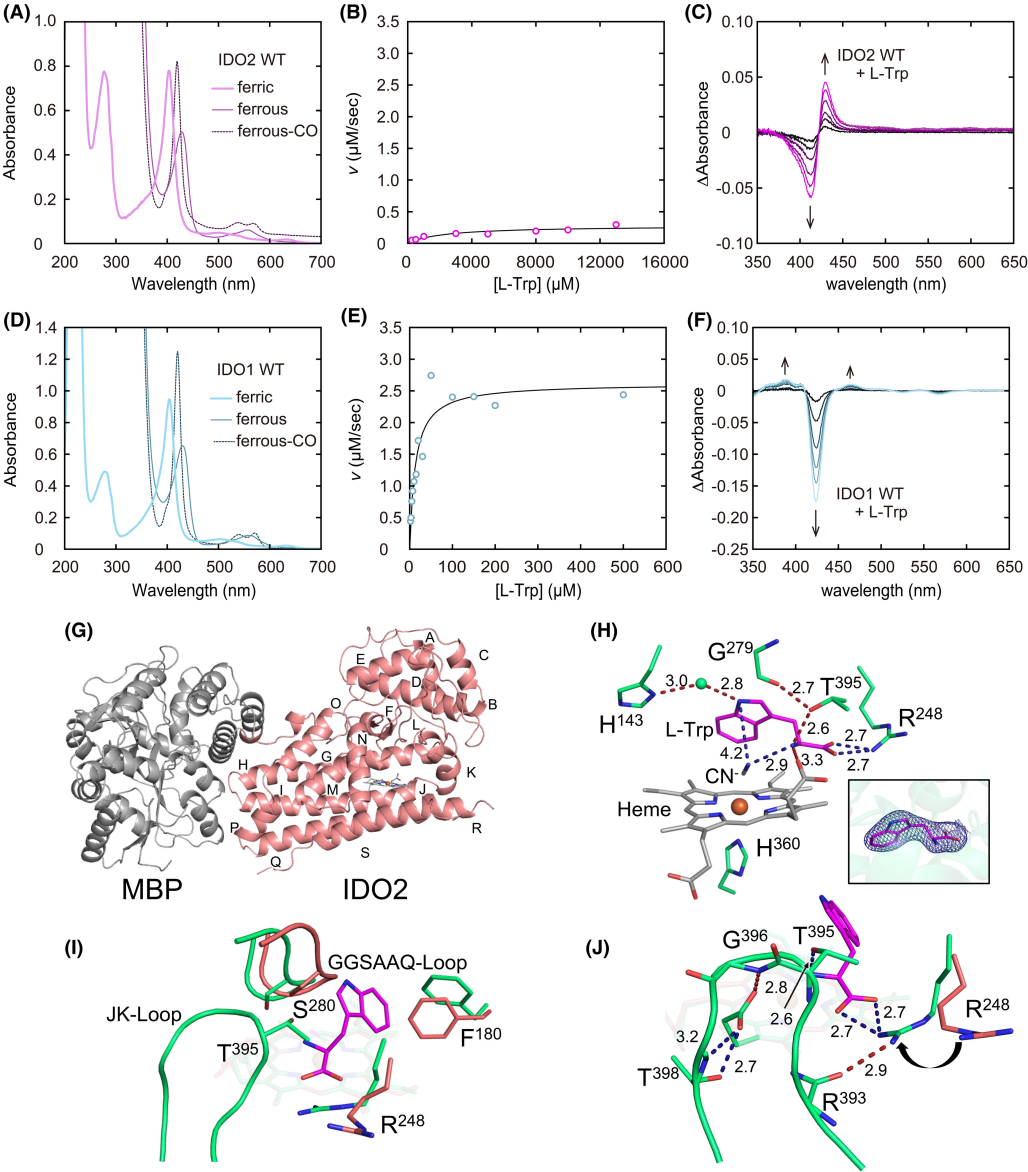
Functional and structural characterization of IDO2. (A) UV–Vis absorption spectra in the ferric (thick line), ferrous (thin line), and ferrous‐CO (dashed line) forms of IDO2 WT. (B) The Michaelis–Menten plot of IDO2 WT when L‐Trp was used as a substrate. (C) Differential spectra of IDO2 WT titrated with L‐Trp. (D) UV–Vis absorption spectra in the ferric (thick line), ferrous (thin line), and ferrous‐CO (dashed line) forms of IDO1 WT. (E) The Michaelis–Menten plot of IDO1 WT when L‐Trp was used as the substrate. (F) Differential spectra of IDO1 WT titrated with L‐Trp. (G) The overall structure of MBP‐IDO2 in the resting state. The helices of the IDO2 moiety (helices A‐S) are named in the order of appearance in the primary sequence. (H) The active site of IDO2 in complex with CN^−^ and L‐Trp. The iron atom and a water molecule are illustrated by brown and green spheres, respectively. The inset shows the sigma‐A‐weighted 2*F*_o_–*F*_c_ map for L‐Trp contoured at 1.0σ. (I) Superimposition of the resting (salmon) and the L‐Trp complex (green) states of IDO2. (J) Structural changes around the active site and hydrogen bonds involved in L‐Trp binding. Nitrogen and oxygen atoms are colored blue and red, respectively. Distances are shown as dotted lines, with values given in Å. Structural figures were generated by PyMOL (https://www.pymol.org/).

In this study, we used human IDO2 with the N‐terminal 13 residues deleted. This construct is referred to as IDO2 wild‐type (IDO2 WT) throughout this manuscript. The IDO2 WT protein was purified, and its characterization was performed by measuring its ultraviolet–visible (UV–Vis) absorption spectrum and catalytic activity (Fig. 1A–C). For comparison, we purified IDO1 WT protein and performed the same analyses (Fig. 1D–F).

The Soret band peaks of ferric IDO2 and IDO1 were observed at 403.5 and 404.5 nm, respectively (Fig. 1A,D). In addition, the spectra of the ferrous and ferrous–carbon monoxide (ferrous–CO) forms of IDO2 showed only slight shifts of approximately 1–2 nm compared to those of IDO1, indicating that the heme environments of IDO1 and IDO2 are generally similar. The L‐Trp metabolic activity of IDO2 was subsequently measured, and enzymatic parameters were evaluated. The turnover number (*k*_cat_) for IDO2 was 0.239 s^−1^, approximately 1/10 that of IDO1 (Fig. 1B,E, Table 1). In contrast, the Michaelis constant (*K*_m_) value was determined to be 2100 μm, which was approximately 260 times larger than that of IDO1 (7.90 μm). The intrinsic clearance (*CL*_int_ = *k*_cat_/*K*_m_) was extremely low for IDO2 (1.25 × 10^−4^ μm ^−1^·s^−1^). It was approximately 1/2500 of that of IDO1 (3.14 × 10^−1^ μm ^−1^·s^−1^). Although these values are slightly better than those measured in previous reports [12, 41, 42], they confirmed that purified IDO2 exhibited low L‐Trp metabolic activity compared to IDO1.

**Table 1:** Enzymatic activity and binding affinity of human IDO1 and IDO2.

	*K*_m_ (μm)	*k*_cat_ (s^−1^)	*CL*_int_ (μm ^−1^·s^−1^)	*K*_d_ (μm)
IDO2 WT	2100 ± 725	0.239 ± 0.037	0.000125 ± 0.000034	122.2 ± 3.6
IDO2^a^	6809 ± 917	0.103 ± 0.006	0.000015 ± 0.000022	–
IDO2^b^	9360 ± 810	0.0933 ± 0.0117	0.00001 ± 0.0000015	–
IDO2^c^	–^d^	–^d^	0.0045	–
MBP‐IDO2 WT	1792 ± 66	0.390 ± 0.091	0.000220 ± 0.000059	66.6 ± 5.4
IDO1 WT	7.90 ± 3.58	2.17 ± 0.37	0.314 ± 0.102	37.9 ± 2.3
IDO1^a^	22 ± 2	10.9 ± 0.27	0.496 ± 0.047	–
IDO1^c^	14.1 ± 0.44	1.00 ± 0.08	0.0709 ± 0.0061	–

^a^ Ref. [41].

^b^ Ref. [42].

^c^ Ref. [12].

^d^ Saturation not observed at L‐Trp concentrations tested.

To further characterize the binding affinity of IDO2 for L‐Trp, we monitored changes in the UV–Vis absorption spectra. To evaluate binding affinity, potassium cyanide was used to obtain a stable ferric–cyanide complex, which mimics the activated IDO form bound to O_2_^−^. Because the cyanide ion (CN^−^) has a size and charge similar to O_2_^−^, it has been used as a surrogate of dioxygen ligand for structural analyses of IDO1 [43, 44]. However, it should also be noted that the CN^−^ complex is not catalytically active, and thus, the *K*_d_ values obtained under these conditions represent an approximation of substrate affinity in a surrogate state.

The difference spectrum of IDO2 upon L‐Trp binding showed a sinusoidal shape, with a minimum at approximately 413 nm and a maximum near 430 nm (Fig. 1C). This spectral change was completely different from that observed upon L‐Trp binding to IDO1 (Fig. 1F) and was more similar to the difference spectrum observed when D‐Trp binds to IDO1 (Fig. S1). Because IDO1 exhibits extremely low metabolic activity with D‐Trp, this type of spectral change is indicative of a low‐productive state at the catalytic site. The spectral changes were analyzed and the values of the dissociation constants (*K*_d_) were assessed. In IDO2, the *K*_d_ value for L‐Trp was 122.2 μm, approximately three times higher than that of IDO1 (37.9 μm) (Table 1). This result indicates that although the binding mode differs markedly, IDO2 still binds to L‐Trp with a relatively high affinity, and the difference in *K*_d_ values is not as pronounced as that observed in the metabolic activity.

### Crystal structure of IDO2

The lack of an experimentally determined structure of IDO2 has hampered detailed investigation of the structure–function relationship of IDO2. Therefore, we performed X‐ray crystallographic analysis of IDO2. Because the IDO2 WT protein is unstable in the absence of a high concentration of glycerol, which is necessary for stabilization of the protein but can inhibit crystallization, we prepared a more stable recombinant IDO2 containing an N‐terminal MBP‐tag (MBP‐IDO2).

MBP‐IDO2 showed spectroscopic and enzymatic properties (*K*_d_, *K*_m_, *CL*_int_ for L‐Trp: 66.6 μm, 1792 μm, and 2.20 × 10^−4^ μm ^−1^·s^−1^, respectively) similar to those of IDO2 (Fig. S2, Table 1). The slightly better parameters observed for MBP‐IDO2 are likely attributable to the higher purity of the crystallization‐grade protein sample. Nevertheless, because the biochemical properties of the MBP‐fused construct closely match those of IDO2 without a tag, we consider that structural analyses performed using the MBP‐tagged protein can be reliably used to interpret and discuss the biochemical behavior of IDO2.

First, we determined a resting‐state structure of MBP‐IDO2 at 2.45 Å resolution (Table 2, Fig. 1G). There is one MBP‐IDO2 molecule in the asymmetric unit that exhibited a monomeric state. IDO1 often dimerizes via Cys308, which forms a disulfide bond with a neighboring protomer [43]. In contrast, IDO2 lacks this Cys residue and remains monomeric. IDO2 is composed of a small N‐terminal domain (helices A–E) and a large C‐terminal domain (helices F–S) containing one heme molecule. The iron atom of the heme is coordinated by H360 on helix Q, which corresponds to H346 in IDO1. The overall structure of the IDO2 moiety in MBP‐IDO2 is similar to the structure of IDO1 (C^α^ RMSD = 0.85 Å). Electron density of the loop region between helices J and K (JK‐loop) was not observed, indicating that this loop was disordered, similar to the corresponding loop region in the resting state of IDO1 [44].

**Table 2:** Data collection and refinement statistics for Resting, L‐Trp‐bound, and 5HT‐bound state structures.

	IDO2 WT resting state	IDO2 WT L‐Trp complex	IDO2 WT 5HT complex	IDO2 H143Y L‐Trp complex
Data collection				
Diffraction source	SPring‐8 BL44XU			
Wavelength (Å)	0.9			
Space group	*P*3_1_21			
*a*, *b*, *c* (Å)	198.26, 198.26, 93.59	196.64, 196.64, 93.66	197.64, 197.64, 93.75	196.71, 196.82, 93.61
*α*, *β*, *γ* (°)	90.08, 90.00, 119.99	90.00, 90.00, 120.00	90.00, 90.00, 120.00	90.02, 90.00, 119.98
Resolution (Å)	48.83–2.45 (2.50–2.45)	49.16–2.25 (2.29–2.25)	42.35–2.45 (2.54–2.45)	48.56–2.35 (2.39–2.35)
Total reflections	538 345	685 150	540 017	592 023
Unique reflections	77 694	98 320	77 336	86 600
Completeness (%)	99.9 (100.0)	99.8 (99.3)	99.9 (99.9)	99.9 (99.7)
Redundancy	6.9 (6.4)	7.0 (6.9)	7.0 (7.2)	6.8 (6.4)
*I*/*σ*(*I*)	10.1 (0.7)	11.5 (0.8)	10.8 (0.7)	9.7 (0.6)
CC_1/2_	0.998 (0.548)	0.999 (0.602)	0.999 (0.588)	0.999 (0.583)
*R*_merge_ (all I+ & I−)	0.112 (2.227)	0.096 (3.638)	0.119 (3.298)	0.107 (2.496)
*R*_meas_ (all I+ & I−)	0.122 (2.426)	0.0104 (3.931)	0.128 (3.557)	0.115 (2.719)
Refinement				
Resolution (Å)	45.15–2.45	43.53–2.25	42.35–2.45	45.13–2.35
*R*_work_/*R*_free_ (%)	0.2246/0.2559	0.2721/0.3034	0.2346/0.2668	0.2213/0.2478
Bond length (Å)	0.009	0.010	0.011	0.011
Bond angle (°)	1.16	1.26	1.22	1.37
*B*‐factor (mean) (Å^2^)				
Overall	76.16	69.32	73.25	72.76
Protein	76.17	69.25	73.29	72.86
Water	77.74	72.08	70.58	69.18
Ligand	71.39	67.03	73.65	73.07
Ramachandran plot (%)				
Favored	95.95	96.50	95.66	95.01
Allowed	3.92	3.50	4.34	4.86
Outliers	0.14	0.00	0.00	0.13
PDB code ID	9UZ0	9UYY	9UYZ	9UZ1

Next, we soaked a crystal of apo MBP‐IDO2 in a reservoir solution containing L‐Trp and potassium cyanide and collected the diffraction data. The MBP‐IDO2 structure in complex with L‐Trp and CN^−^ was determined at 2.25 Å resolution (Fig. 1H, Table 2). CN^−^ is observed as a slightly inflated electron density on the iron atom of the heme, and the electron density of L‐Trp is clearly observed above the heme. L‐Trp is stabilized at the active site by hydrogen bonding with R248, T395, and the propionic acid moiety of the heme. The N^ε^ atom of L‐Trp indirectly interacts with the N^δ^ atom of H143 through a water molecule. The indole ring of L‐Trp forms a T‐stack with the phenyl group of F180.

A comparison of the resting‐state and complex structures revealed that the binding of L‐Trp induced structural changes around the catalytic site (Fig. 1I,J). The JK‐loop moves dynamically to form hydrogen bonds with the N atom of the amino group of L‐Trp via T395 and with the propionic acid moiety of the heme through G396 and T398. R248 reorients its side chain to interact with the carboxyl group of L‐Trp. R248 also forms a hydrogen bond with the carbonyl O atom of R393 in the JK‐loop, resulting in fixation of the JK‐loop near the active site. These structural changes in the JK‐loop and R248 (R231 in IDO1) during the binding of L‐Trp have been observed in IDO1 [45].

### Structural comparison of IDO1 and IDO2

**Fig. 2:**
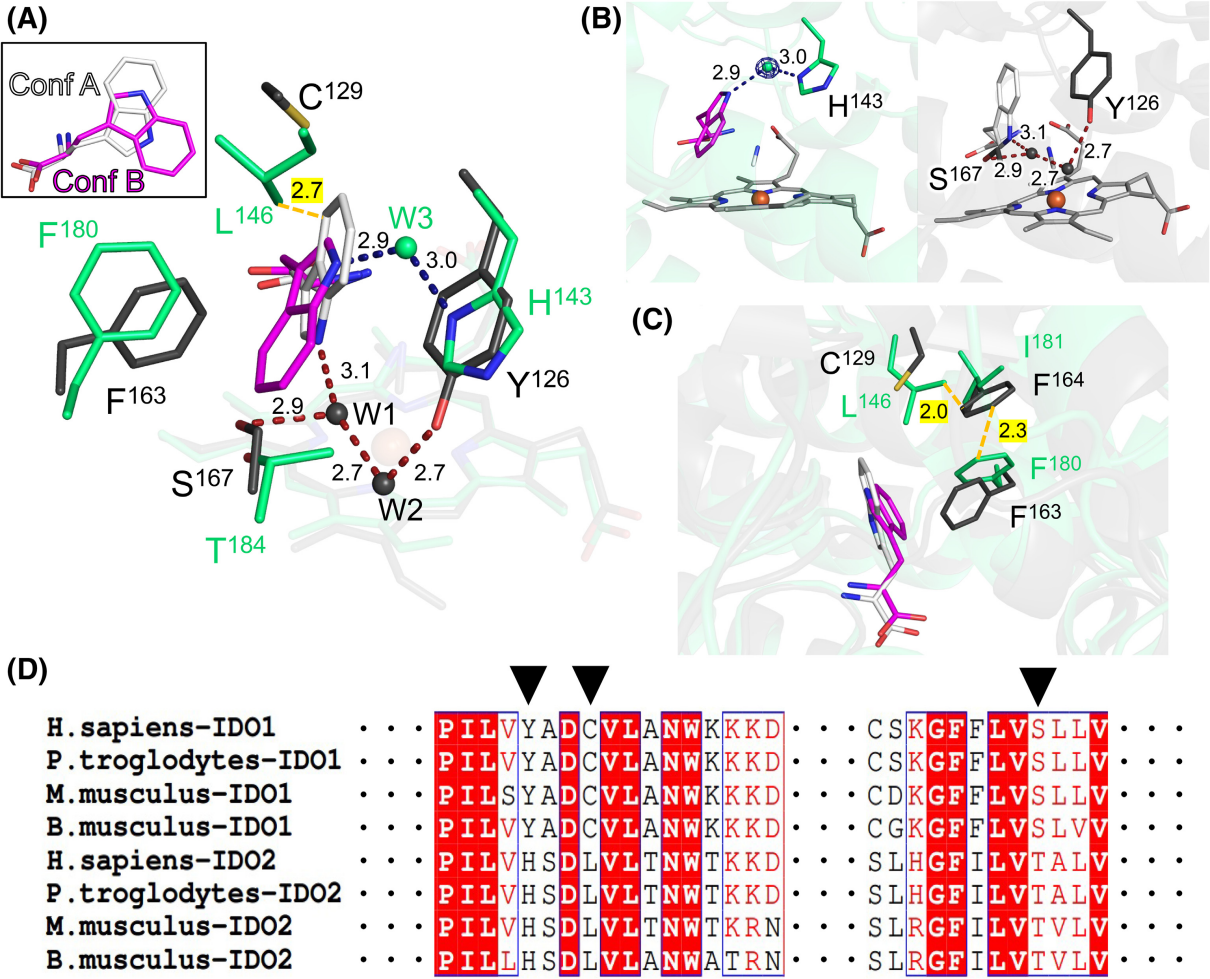
Comparison of binding modes of L‐Trp. (A) Superimposition of the active sites in IDO1 (PDB ID: 5WMU) (gray) and IDO2 (green). Hydrogen bonds are illustrated by dashed lines with distances indicated in Å. An orange dashed line shows the distance between L146 in IDO2 and L‐Trp assuming Conf A observed in IDO1. The inset shows the difference in binding modes of L‐Trp in IDO1 (white) and in IDO2 (magenta). Water molecules of IDO1 and IDO2 are shown by gray and green spheres, respectively. Yellow‐highlighted text indicates the distance between L146 in IDO2 and L‐Trp in IDO1. (B) Hydrogen bond networks of the active site in IDO2 (left) and in IDO1 (right). The sigma‐A‐weighted 2*F*_o_–*F*_c_ map of the water molecule is contoured at 1.0σ. (C) Comparison of residues around L‐Trp. Orange dashed lines show distances between residues in IDO2 and those in IDO1. Conf A and Conf B mean conformations A and B of L‐Trp, respectively. Yellow‐highlighted texts indicate distances between residues in IDO2 (L146 or F180) and F164 in IDO1. Structural figures were generated by PyMOL. (D) Sequence alignment of IDOs. The positions of conserved H143, L146, and T184 are indicated by black arrowheads. UniProt accession numbers used for alignment are as follows: *Homo sapiens* IDO1 (P14902), *H*. *sapiens* IDO2 (Q6ZQW0), *Pan troglodytes* (chimpanzee) IDO1 (H2QW24), *P*. *troglodytes* IDO2 (H2QW25), *Mus musculus* (mouse) IDO1 (P28776), *M. musculus* IDO2 (Q8R0V5), *Balaenoptera musculus* (blue whale) IDO1 (A0A8B8WB37), and *B. musculus* IDO2 (A0A8B8WBD8). Sequence alignments were performed with Clustal Omega (https://www.ebi.ac.uk/jdispatcher/msa/clustalo). Visual representations of the sequence alignments were generated using ESPript 3.0 (http://espript.ibcp.fr).

Comparison of the L‐Trp complex structures of IDO1 and IDO2 revealed differences in the orientation of the indole ring in L‐Trp (Fig. 2A). The conformations of L‐Trp observed in IDO1 and IDO2 were referred to as Conf A and Conf B, respectively. Conf A is also observed in crystal structures of the alternative L‐Trp metabolizing enzyme in humans, TDO (Fig. S3) [46]. The difference in L‐Trp conformation can be attributed primarily to two amino acids unique to IDO2: H143 and L146. H143 can stabilize Conf B through water‐mediated interactions with the indole ring of L‐Trp. H143 is replaced by tyrosine (Y126) in IDO1, preventing the water molecule from being located at a similar position. Instead, a different hydrogen‐bonding network was formed in IDO1. In IDO1, S167 forms a hydrogen bond with a water molecule that interacts with the N^ε^ atom of L‐Trp of Conf A and a nearby water molecule forming a hydrogen bond with Y126 (Fig. 2B). In IDO2, the corresponding residue is replaced by T184 which possesses an additional methyl group, resulting in the expulsion of water molecules that form the aforementioned hydrogen bond network. Therefore, our present structure is consistent with a previous biochemical study showing that the difference in properties between IDO1 and IDO2 can be attributed to Y126 and T184 [38].

L146 in IDO2 is placed at a position showing steric hindrance with L‐Trp in Conf A (Fig. 2A), while the corresponding residue in IDO1 (C129) can stabilize L‐Trp in Conf A via van der Waals interactions. In addition, F164 in IDO1 was replaced by the less bulky I181 in IDO2, creating a space where F180 in IDO2 moved to form an adequate T‐stacking interaction with L‐Trp in Conf B (Fig. 2C). The distance from CN^−^ to the indole ring of L‐Trp in IDO2 is longer than that in IDO1. For example, the distances between the N atom of CN^−^ and the N^ε^ atom of L‐Trp are 4.2 and 3.4 Å in IDO2 and IDO1, respectively (Fig. 1H). Although the crystal structure of TDO shows that the binding mode of CN^−^ on the heme is slightly different from that of a dioxygen ligand (Fig. S4) [46], the longer distance between the axial diatom ligand to the indole ring of the substrate can explain the low catalytic activity of IDO2.

Comparison of amino acid sequences around the catalytic site between IDO1 and IDO2 is shown in Fig. 2D.

### Effect of mutation at the IDO2‐specific residue His143

It was shown that a specific residue of IDO2 plays a critical role in L‐Trp recognition by IDO2. To gain further insight into the substrate recognition of IDO2, we introduced a mutation at His143, which interacts with L‐Trp via a water molecule, by replacing it with the corresponding tyrosine in IDO1 to produce the IDO2 H143Y mutant and evaluated the resulting changes in its properties.

**Fig. 3:**
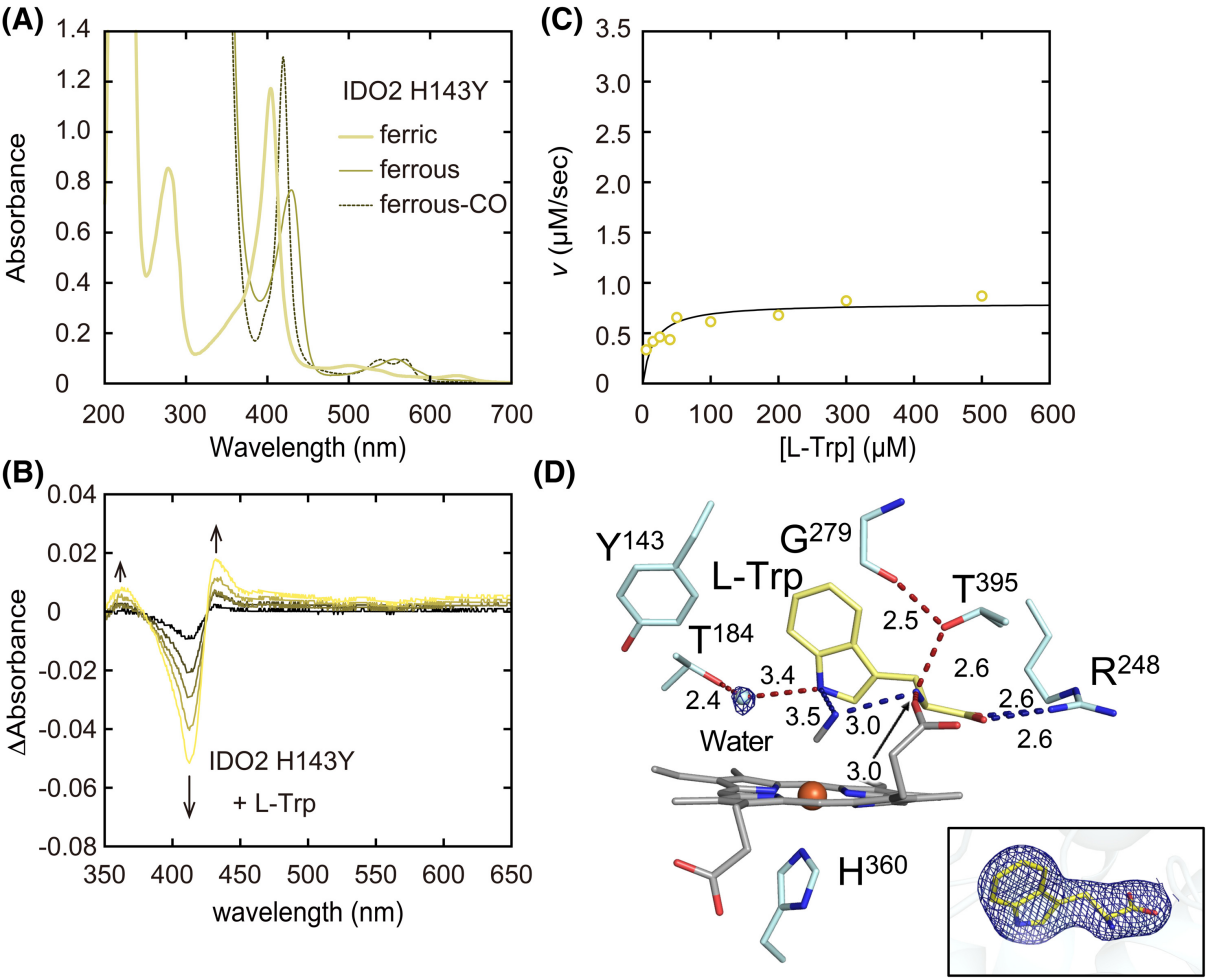
Reactivity of the IDO2 H143Y mutant. (A) UV–Vis absorption spectra in the ferric (thick line), ferrous (thin line), and ferrous‐CO (dashed line) forms of the IDO2 H143Y mutant. (B) Differential spectra of the IDO2 H143Y mutant titrated by L‐Trp. (C) The Michaelis–Menten plot of the IDO2 H143Y mutant when L‐Trp is used as the substrate. (D) The crystal structure of the IDO2 H143Y mutant in complex with L‐Trp. Distances are shown as dotted lines, with values given in Å. The sigma‐A‐weighted 2*F*_o_–*F*_c_ maps of the water molecule and L‐Trp are contoured at 1.0σ. The structural figure was generated by PyMOL.

First, UV–Vis absorption spectroscopy was performed to determine whether the mutation altered the electronic environment around the heme. The Soret band of IDO2 H143Y exhibited a maximum at 404 nm. Additionally, the ferrous and ferrous‐CO form showed absorption peaks at 429.5 nm and 419.5 nm, respectively. These spectral features indicate that IDO2 H143Y retained a heme environment almost identical to that of IDO2 WT and IDO1 WT (Fig. 3A).

Subsequently, the binding characteristics of the H143Y mutant for L‐Trp were evaluated. The *K*_d_ value of IDO2 H143Y for L‐Trp was determined to be 37.5 μm, indicating that its binding affinity was very close to that of IDO1 (Table 3). Furthermore, the difference spectrum of IDO2 H143Y shifted toward a profile similar to that of IDO1 WT (Figs 1F, 3B). Specifically, while IDO2 WT exhibited a sinusoidal difference spectrum with a minimum at 413 nm and a maximum at 430 nm with comparable amplitudes, the H143Y mutant displayed a similar wavelength pattern but with an altered amplitude. When analyzed using molar extinction coefficients (*ε*), the *ε* value at 430.5 nm for IDO2 WT increased from 58.9 to 66.3 upon L‐Trp binding, corresponding to a 1.12‐fold increase. In contrast, the *ε* at 433.5 nm for IDO2 H143Y increased only slightly from 49.9 to 52.1, a 1.04‐fold change. These results indicate that although the wavelengths remained similar, the sinusoidal difference spectrum observed for IDO2 WT shifted to a more trough‐shaped pattern upon His143 mutation. Additionally, in IDO2 H143Y, a new absorption peak emerged at approximately 362 nm upon L‐Trp binding, resembling the 385 nm peak observed in IDO1 upon L‐Trp binding. These spectral changes suggest that the heme environment of IDO2 H143Y in the presence of L‐Trp more closely resembles that of IDO1 WT.

**Table 3:** Enzymatic activity and binding affinity of the H143Y mutant of IDO2 for L‐Trp.

	IDO2 H143Y	Ratio (/IDO2 WT)	Ratio (/IDO1 WT)
*K*_m_	5.60 ± 1.88 (μm)	0.00267	0.709
*k*_cat_	0.758 ± 0.299 (s^−1^)	3.17	0.349
*CL*_int_	0.161 ± 0.080 (μm ^−1^·s^−1^)	1290	0.513
*K*_d_	37.6 ± 5.4 (μm)	0.307	0.989
*K*_d_ for 5HT	8880 ± 410 (μm)	34.2	–^a^

^a^ *K*_d_ of IDO1 WT for 5HT could not be measured as shown in Table 4.

To determine whether the mutation is reflected in the catalytic function, we next evaluated the metabolic activity toward L‐Trp (Table 3, Fig. 3C). The results showed a *K*_m_ value of 5.60 μm, a *k*_cat_ value of 0.758 s^−1^, and a *CL*_int_ value of 0.161 μm ^−1^·s^−1^. Notably, IDO2 H143Y exhibited a marked increase in L‐Trp metabolic activity compared to IDO2 WT. The catalytic efficiency (*CL*_int_) of the H143Y mutant was 1290‐fold higher than that of IDO2 WT and reached approximately half the value observed for IDO1, which agrees with an earlier mutation study [38]. These enzymatic results demonstrated that mutating His143 to tyrosine results in IDO1‐like properties.

We then conducted crystallographic analysis of the H143Y mutant (Table 2, Fig. 3D). The structure of MBP‐IDO2 H143Y in complex with L‐Trp and CN^−^ was determined at a resolution of 2.35 Å. Our structural analysis revealed that the binding mode of L‐Trp in the H143Y mutant was Conf A, as observed in IDO1, and not Conf B, as observed in IDO2 WT. This is because Tyr143 in IDO2 is unable to interact with the N^ε^ atom of L‐Trp in Conf B via a water molecule. The distance between CN^−^ and the N^ε^ atom of the indole ring of L‐Trp in IDO2 H143Y (3.5 Å) is almost identical to that in IDO1 (3.4 Å). These structural observations explain the significantly higher catalytic turnover rate of IDO2 H143Y, which is comparable to that of IDO1.

In IDO1, the N^ε^ atom of L‐Trp forms a hydrogen‐bonding network via two water molecules, extending to Y126, E171, and S267. The first water molecule (W1) (Fig. 2A), which forms hydrogen bonds with the N^ε^ atom of L‐Trp, is stabilized by S167. However, in IDO2, the corresponding residue is replaced by T184, which possesses an additional methyl group, resulting in the expulsion of water molecules (W2) that form the above‐mentioned hydrogen bond network. Although the precise functional role of the active‐site water molecules has not been fully elucidated, previous studies suggest that they help shape the electrostatic environment around the ligand and are also important for maintaining the oxyanion hole that stabilizes the anionic intermediate formed after O–O bond cleavage [47]. Therefore, the disrupted hydrogen‐bonding network in IDO2‐H143Y (Fig. 3D) may explain the lower activity of H143Y toward L‐Trp compared to that of IDO1.

### Evaluation of the binding properties of tryptophan analogs to IDO2

As described above, we thoroughly investigated the interaction between IDO2 WT and L‐Trp and elucidated the underlying reasons for its low metabolic activity. Building on these findings, we aimed to further characterize the binding properties of IDO2 WT using various tryptophan analogs.

First, we evaluated the binding affinities of IDO2 WT to eight tryptophan analogs: 1‐methyl L‐tryptophan (L‐1MT), an IDO1 inhibitor which has a methyl group at the 1‐position; 5‐hydroxy‐tryptophan (5HTP), with a hydroxyl group introduced at the 5‐position; D‐tryptophan (D‐Trp), a stereoisomer; tryptamine (TRY), which lacks the carboxyl group on the main chain; 3‐indolepropionic acid (3IPA), which lacks the amino group on the main chain; serotonin (5HT), which has a hydroxyl group introduced at the 5‐position of TRY; 5‐methyl‐l‐tryptophan (L‐5MT); and 5‐methoxy‐l‐tryptophan (L‐5MoT), which has a modifying group introduced at the 5‐position. The compound structures and obtained *K*_d_ values are listed in Table 4.

**Table 4:** Binding affinity *K*_d_ (μm) of WT IDO1 and IDO2 for L‐Trp and its analogs.

	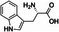 L‐Trp	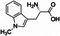 L‐1MT	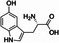 5HTP	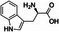 D‐Trp	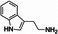 TRY	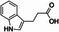 3IPA	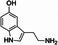 5HT	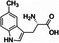 L‐5MT	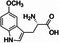 L‐5MoT
IDO2	122.2 ± 3.6	15.6 ± 3.9	1920 ± 211	517 ± 24	1870 ± 219	N.D.	260 ± 38	66.0 ± 4.5	35.2 ± 2.7
IDO1	37.9 ± 2.3	4.3 ± 0.9	1360 ± 74	7180 ± 907	4540 ± 713	N.D.	N.D.	26.2 ± 2.8	57.7 ± 1.3

L‐1MT, which exhibited stronger binding to IDO1 WT than L‐Trp, also bound strongly to IDO2 WT, with a *K*_d_ of 15.6 μm. In contrast, 5HTP, in which a hydroxyl group is introduced at the 5‐position of the indole ring, showed markedly weaker binding (*K*_d_ = 1920 μm). The mirror isomer, D‐Trp, exhibited a higher *K*_d_ (517 μm) than that of L‐Trp, yet still displayed considerably stronger binding than the corresponding value for IDO1 WT with D‐Trp (*K*_d_ = 7180 μm), suggesting that IDO2 WT possesses relatively low chiral selectivity toward tryptophan. The *K*_d_ values of TRY, which lacks the carboxyl group on the main chain, and 5HT, which is hydroxylated at the 5‐position, were 1870 μm and 260 μm, respectively. Notably, 5HT induced no measurable spectral changes upon incubation with IDO1 and the *K*_d_ value could not be obtained. The *K*_d_ value of H143Y IDO2 for 5HT was also determined to be 8880 μm, which is approximately 34 times worse than that of IDO2 WT (Table 3), suggesting that H143 unique to IDO2 is involved in recognition of 5HT. Furthermore, 3IPA, which does not have an amino group, did not bind well to either IDO1 or IDO2, and the *K*_d_ value could not be determined. Finally, L‐5MT and L‐5MoT, bearing methyl and methoxy groups at the 5‐position, respectively, exhibited relatively strong binding affinities, with *K*_d_ values of 66.0 μm and 35.2 μm, respectively. Compared to 5HTP, which also features a 5‐position modification, these compounds showed dissociation constants more similar to those of L‐Trp. This observation suggests that the polarity of the substituent at the 5‐position significantly influences binding affinity. Collectively, these results indicate that IDO2 may accommodate a greater structural diversity of ligands than IDO1, reflecting broader ligand‐binding tolerance in its binding pocket.

D‐Trp, TRY, 5HT, and L‐5MoT bind more strongly to IDO2 than to IDO1. Among these, 5HT appears to be unique because its binding to IDO1 was not observed in this study. We measured the enzymatic activity of these compounds. No activity was detected for D‐Trp or 5HT, whereas both L‐5MoT (2.69 × 10^−3^ μm ^−1^·s^−1^; 21.5‐fold higher than with L‐Trp) and L‐5MT (1.37 × 10^−2^ μm ^−1^·s^−1^; 109.6‐fold higher than with L‐Trp) exhibited higher *CL*_int_ than L‐Trp (Table 5). These values are approximately one or two orders of magnitude greater than those obtained with the corresponding racemic mixtures (5‐methyl‐D,L‐tryptophan [D,L‐5MT] and 5‐methoxy‐D,L‐tryptophan [D,L‐5MoT]) [41], indicating that IDO2 is capable of recognizing these substrates in a stereoselective manner.

**Table 5:** Enzymatic parameters of IDO2 WT and IDO2 H143Y for L‐Trp analogs.

IDO2	*K*_m_ (μm)	*k*_cat_ (s^−1^)	*CL*_int_ (μm^−1^ ·s^−1^)
WT 5HT	N.D.	N.D.	N.D.
H143Y 5HT	N.D.	N.D.	N.D.
WT D‐Trp	N.D.	N.D.	N.D.
WT 5‐HTP	N.D.	N.D.	N.D.
WT L‐5MT	44.6 ± 22.3	0.449 ± 0.017	0.0137 ± 0.0076
WT L‐5MoT	311 ± 142	0.648 ± 0.135	0.00269 ± 0.00133
H143Y L‐5MT	289 ± 186	0.890 ± 0.362	0.00498 ± 0.00302
H143Y L‐5MoT	25.6 ± 22.4	0.237 ± 0.030	0.0223 ± 0.01696

We also performed activity assays of the H143Y mutant using 5HT, L‐5MT, and L‐5MoT as substrates (Table 5). IDO2 H143Y metabolized L‐5MT and L‐5MoT while it did not show activity toward 5HT. The *CL*_int_ value for L‐5MT (4.98 × 10^−3^ μm ^−1^·s^−1^) was approximately half of that of the WT protein, indicating that substitution of H143 with IDO1‐specific tyrosine does not markedly affect binding of this compound. In contrast, the H143Y mutant showed approximately one order of magnitude higher catalytic activity for L‐5MoT (2.23 × 10^−2^ μm ^−1^·s^−1^) compared with the WT enzyme. The small *K*_m_ value of 25.6 μm may contribute to this one‐order‐of‐magnitude difference in catalytic activity. However, because the associated error (± 22.4 μm) is large, it is possible that the *K*_m_ value was not estimated with sufficient precision.

### Crystal structures of IDO2 in complex with tryptophan derivatives

**Fig. 4:**
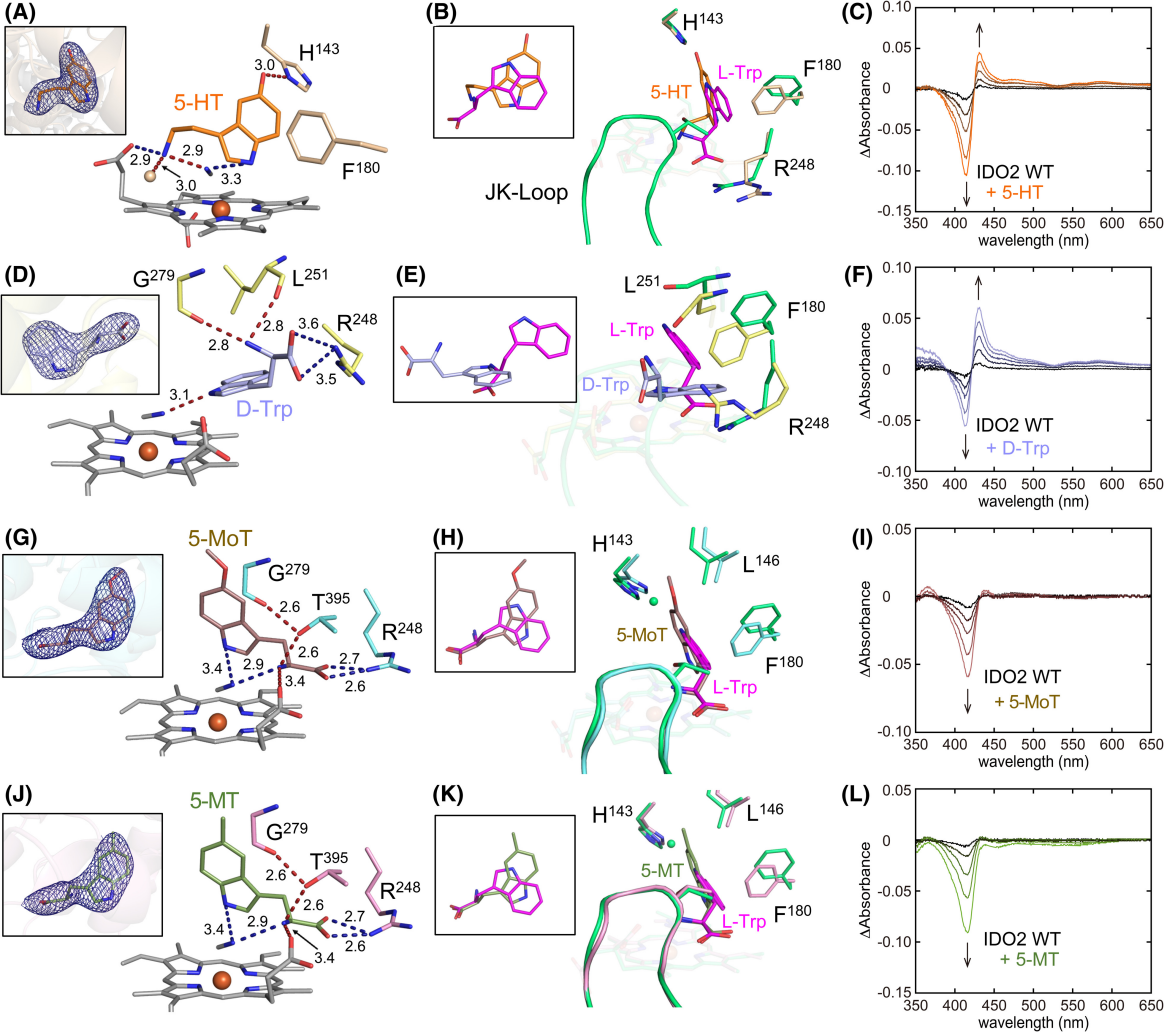
Structural and spectroscopic analysis of IDO2 in complex with L‐Trp analogs. (A) The crystal structure of the IDO2–5HT complex. (B) Structural comparison of the IDO2–5HT complex (light beige) with the IDO2–L‐Trp complex (green). (C) Differential spectra of IDO2 WT titrated with 5HT. (D) The crystal structure of the IDO2–D‐Trp complex. (E) Structural comparison of the IDO2–D‐Trp complex (yellow) with the IDO2–L‐Trp complex (green). (F) Differential spectra of IDO2 WT titrated with D‐Trp. (G) The crystal structure of the IDO2–5MoT complex. (H) Structural comparison of the IDO2–5MoT complex (light blue) with the IDO2–L‐Trp complex (green). (I) Differential spectra of IDO2 WT titrated with 5MoT. (J) The crystal structure of the IDO2–5MT complex. (K) Structural comparison of the IDO2–5MT complex (pink) with the IDO2–L‐Trp complex (green). (L) Differential spectra of IDO2 WT titrated with 5MT. The sigma‐A‐weighted 2*F*_o_–*F*_c_ map around ligands is contoured at 1.0σ. Water molecules are illustrated by small spheres. Distances are shown as dotted lines, with values given in Å. These structural figures were generated by PyMOL.

To further elucidate the structural basis of reactivity and stereoselectivity for tryptophan derivatives, we performed structural analyses. The crystal structure of MBP‐IDO2 in complex with 5HT and CN^−^ was determined at 2.45 Å resolution (Table 2). The most prominent difference between L‐Trp and 5HT bound to IDO2 was in the direction of the indole rings (Fig. 4A,B). The indole ring of 5HT is flipped compared to that of L‐Trp observed in the L‐Trp complex of IDO2. Consequently, the position of F180, which forms a T‐stack with the indole ring of the ligands, is different between the L‐Trp and 5HT complexes. The amino and hydroxyl groups of 5HT form hydrogen bonds with the propionate moieties of the heme and H143, respectively. In contrast to L‐Trp, 5HT, which lacks a carboxyl group, does not interact with R248. Consequently, R248 showed no structural changes, and the JK‐loop remained at the same position as that observed in the resting state (Fig. 4B). This result suggests that the movement of R248 through its interaction with the carboxyl group of the ligand induces the displacement of the JK‐loop. In the case of 5HT, the indole ring was positioned close to that observed in Conf A, and the difference spectrum exhibited a relatively low ε around the absorption maximum, with a shape approaching a concave‐down curve (Fig. 4C). However, since metabolic activity was not detected and the indole ring was slightly displaced, this nonproductive binding mode was designated as Conf A′. In Conf A′, the N^ε^ atom on the indole ring does not seem to form a hydrogen bond with a nearby water molecule, because it faces the heme plane.

We also determined the crystal structure of MBP‐IDO2 in complex with 5HTP at 2.68 Å resolution (Table 6, Fig. S5A,B). The only structural difference between 5HT and 5HTP is that 5HTP possesses a carboxyl group. As expected, the carboxyl group of 5HTP interacts with R248; however, compared with the L‐Trp complex data, the electron density of the JK‐loop was poorly defined, indicating that the rearrangement of the JK‐loop position was not fully completed (Fig. S5C,D). The binding conformation of 5HTP is more similar to that of 5HT than that of L‐Trp in Conf A because its hydroxyl group and H143 possibly form a hydrogen bond, although the distance between the hydroxyl group of 5HTP and H143 (3.6 Å) is slightly longer than typical hydrogen bond distances and the binding position of 5HTP slightly shifts compared to 5HT. This may be because the carboxyl and amino groups of 5HTP retain interactions with R248 and the JK‐loop, respectively, whereas the hydroxyl group cannot simultaneously form a stable hydrogen bond with H143, resulting in an energetically unfavorable conformation. The electron density around the indole ring of 5HTP was unclear (Fig. S5A), suggesting that its binding is not well stabilized. In fact, the *K*_d_ value of 5HTP was more than 7 times worse than that of 5HT (Table 4) and no activity toward 5HTP was observed (Table 5), which agrees with a previous report [41].

**Table 6:** Data collection and refinement statistics for CN‐bound, D‐Trp‐bound, L‐5MT, and L‐5MoT‐bound state structures.

	IDO2 WT CN^−^	IDO2 WT D‐Trp complex	IDO2 WT L‐5MoT complex	IDO2 WT L‐5MT complex	IDO2 WT 5HTP complex	IDO2 H143Y L‐5MoT complex	IDO2 H143Y L‐5MT complex
Data collection							
Diffraction source	SPring‐8 BL44XU						
Wavelength (Å)	0.9						
Space group	*P*3_1_21						
*a*, *b*, *c* (Å)	197.50, 197.50, 93.63	197.38, 197.38, 93.70	198.25, 198.25, 94.11	196.96, 196.96, 93.54	197.96, 197.96, 93.92	198.83, 198.83, 94.26	199.11, 199.11, 94.46
*α, β, γ* (°)	90.00, 90.00, 120.00	90.08, 90.00, 120.00	90.00, 90.00, 120.00	90.00, 90.00, 120.00	90.00, 90.00, 120.00	90.00, 90.00, 120.00	90.00, 90.00, 120.00
Resolution (Å)	49.38–2.55 (2.61–2.55)	49.35–2.50 (2.55–2.50)	49.56–2.50 (2.55–2.50)	48.59–2.50 (2.55–2.50)	48.82–2.68 (2.75–2.68)	49.02–2.60 (2.66–2.60)	49.10–2.55 (2.61–2.55)
Total reflections	472 400	421 798	5 132 260	505 437	414 472	451 080	490 058
Unique reflections	31 635	72 453	73 530	71 852	59 405	65 917	70 117
Completeness (%)	100.0 (100.0)	99.7 (99.4)	99.9 (99.6)	99.5 (99.3)	99.7 (99.6)	99.9 (99.7)	98.8 (99.4)
Redundancy	6.9 (6.9)	5.8 (6.0)	7.0 (7.2)	7.0 (7.2)	7.0 (7.3)	6.8 (7.1)	7.0 (7.3)
*I/σ(I)*	10.9 (0.8)	9.6 (0.4)	15.1 (0.8)	16.8 (1.1)	10.7 (0.7)	10.7 (0.7)	12.2 (0.7)
CC_1/2_	0.998 (0.568)	0.999 (0.517)	0.999 (0.525)	1.000 (0.776)	0.999 (0.540)	0.999 (0.506)	0.998 (0.609)
*R*_merge_ (all I+ & I−)	0.111 (3.220)	0.135 (4.344)	0.075 (3.130)	0.065 (1.340)	0.120 (2.203)	0.115 (2.641)	0.119 (3.767)
*R*_meas_ (all I+ & I−)	0.121 (3.482)	0.149 (4.761)	0.081 (3.371)	0.070 (1.440)	0.129 (2.364)	0.125 (2.862)	0.128 (4.048)
Refinement							
Resolution (Å)	48.7–2.55	42.42–2.50	42.25–2.50	42.25–2.50	45.29–2.68	49.02–2.60	49.10–2.55
*R*_work_/*R*_free_ (%)	0.2066/0.2388	0.2405/0.2746	0.3652/0.3702	0.2009/0.2293	0.2957/0.3321	0.2768/0.3144	0.2091/0.2334
Bond length (Å)	0.012	0.010	0.011	0.010	0.010	0.009	0.009
Bond angle (°)	1.31	1.37	1.18	1.14	1.12	1.05	1.13
*B*‐factor (mean) (Å^2^)							
Overall	78.25	85.54	85.78	87.48	83.67	80.23	81.17
Protein	78.30	85.67	85.86	87.66	83.75	80.30	81.34
Water	73.85	81.98	82.64	79.38	80.77	81.57	72.21
Ligand	782.35	83.06	85.08	85.89	79.81	75.72	75.40
Ramachandran plot (%)							
Favored	95.39	95.82	96.11	96.23	92.33	96.38	96.11
Allowed	4.61	3.78	3.76	3.64	7.40	3.49	3.75
Outliers	0.00	0.40	0.13	0.13	0.27	0.13	0.13
PDB code ID	9UZ2	9UZ3	9UZ4	9UZ5	21OM	21OK	21OO

The crystal structure of MBP‐IDO2 in complex with D‐Trp and CN^−^ was determined at a resolution of 2.50 Å (Table 6, Fig. 4D,E). In this crystal structure, D‐Trp was not observed at the substrate‐binding site, but near the entrance of the substrate pocket. Consequently, the JK‐loop did not move. The difference in binding positions was consistent with the low affinity of D‐Trp. If D‐Trp were positioned at the same site as that of L‐Trp, its C^α^ atom would show steric hindrance with T385, and the amino group would be unable to form hydrogen bonds with T385 owing to its orientation (Fig. S6). This structural constraint may explain why D‐Trp does not bind to IDO2 in the same manner as L‐Trp. The carboxyl group of D‐Trp formed hydrogen bonds with R248. The amino group of D‐Trp formed hydrogen bonds with L251 and G279. Additionally, the N^ε^ atom of D‐Trp directly formed hydrogen bonds with the N atom of CN^−^ (Fig. 4D). In this case, D‐Trp was bound at a position distinct from those observed in Conf A and B, instead of adopting a Conf C binding mode. Conf C is a nonproductive binding mode, which agrees with the spectroscopic data showing a sinusoidal shape (Fig. 4F).

The crystal structures of MBP‐IDO2 in complex with CN^−^ and L‐5MT or L‐5MoT were determined at 2.50 Å resolution (Table 6). For the latter structure, we used a racemic mixture (D,L‐5MoT) during the soaking step; however, we only observed the L‐isomer at the substrate‐binding site. Both L‐5MT and L‐5MoT bound to IDO2 in a manner similar to Conf A of L‐Trp in IDO1, and their titration difference spectra exhibited patterns similar to those observed for L‐Trp binding to IDO1 (Fig. 4G–L). Moreover, movements of the JK‐loop and R248 were observed.

A previous study reported that IDO1 cannot metabolize D,L‐5MoT [41]. This observation led us to hypothesize initially that H143, which is uniquely conserved in IDO2, is a key residue for the binding of L‐5MoT. However, H143 did not interact directly with L‐5MoT. This structural observation is consistent with the result of the activity assay that the H143Y mutant could metabolize L‐5MoT (Table 5). To further confirm that the H143Y mutation does not substantially affect recognition of L‐5MT and L‐5MoT, we determined the H143Y mutant structure in complex with L‐5MT or L‐5MoT at 2.55 and 2.60 Å resolution, respectively (Table 6). The mutation did not significantly alter binding of L‐5MT and L‐5MoT at the catalytic site (Fig. S7).

When comparing IDO1 and IDO2 (Fig. 2A), IDO2‐specific L146 appeared to cause steric hindrance with L‐Trp in Conf A. However, in the L‐5MoT and L‐5MT complex structures, L146 moved slightly and stabilized L‐5MoT or L‐5MT through van der Waals contacts (Fig. S8A,B). In contrast, if L‐Trp were to bind in Conf A, it would be expected to make fewer van der Waals contacts with L146 (Fig. S8C,D), which would likely be insufficient to offset the steric hindrance imposed by this residue. L146 was replaced by C129 in IDO1, which may have caused steric hindrance with the methoxy group of L‐5MoT in Conf A (Fig. S8E,F). This is because the conformation of C129 is restricted by the nearby F164, the corresponding residue of which is the less bulky I181 in IDO2. Therefore, IDO2 has a substrate‐binding site that is more suitable for L‐5MT and L‐5MoT.

## Discussion

### Structural basis of the low catalytic activity of IDO2

Because all our biochemical analyses and structural determinations were conducted by using N‐terminal deleted or MBP‐tag‐fused proteins, future studies on the full‐length protein will be needed to confirm whether catalytic properties and the ligand‐binding conformations observed in this study are relevant under physiological conditions. However, we consider that the constructs used here reflect the physiological state to a sufficient extent to allow meaningful interpretation of the catalytic and structural properties.

IDO2 exhibited extremely low metabolic activity toward L‐Trp, with a *CL*_int_ value of approximately 1/2500 of that of IDO1. Although the overall heme environment and coordination states of IDO1 and IDO2 were similar, our crystallographic analysis revealed that the flipped orientation of the L‐Trp indole ring in IDO2 is a key structural feature underlying its low activity. This flipped orientation, stabilized by the IDO2‐specific residue His143 via a water‐mediated interaction, results in a larger distance between the C2 and C3 bond of L‐Trp and the CN^−^ ligand (a mimic of O_2_^−^), likely decreasing the efficiency of oxygen insertion. The resulting sinusoidal difference spectrum observed for IDO2 further supports the presence of a distinct, catalytically unfavorable L‐Trp conformation.

The introduction of the H143Y mutation reversed the binding mode to Conf A (as seen in IDO1), resulting in an approximately 1300‐fold increase in catalytic efficiency. However, the activity of the H143Y mutant was slightly lower than that of IDO1. This residual difference may be explained by the absence of a complete hydrogen bond network at the catalytic site. In IDO1, S167 stabilizes a water molecule connected to the hydrogen‐bonding network. In IDO2, the corresponding residue (T184) contains an additional methyl group that disrupts the water‐mediated network. Such differences in the organization of active‐site water molecules may contribute to the distinct reactivity observed between IDO1 and IDO2. These key residues, T184 and H143, are conserved among vertebrate IDO2s [38, 48]. Additionally, catalytic reactivity may be influenced by subtle structural differences that are not readily discernible at the current resolution of the available IDO1 and IDO2 crystal structures. Collectively, our results demonstrate that IDO2 possesses a conserved structural feature that limits activity, with His143 playing a central role.

### Expanded substrate recognition of IDO2 and its physiological relevance

IDO2 showed unexpected catalytic activity toward L‐5MT and L‐5MoT. Both substrates adopted Conf A at the active site, mimicking the productive binding mode observed in the IDO1–L‐Trp complex. These compounds seem to be better accommodated in IDO2 than L‐Trp, especially with van der Waals interactions with L146, an IDO2‐specific residue. Compared with IDO1, IDO2 shows markedly enhanced activity toward L‐5MT and L‐5MoT. The catalytic efficiency *k*_cat_/*K*_m_ for L‐5MT is comparable to 0.011 μm ^−1^·s^−1^ of human TDO for L‐Trp [49]. Moreover, it is of the same order of magnitude as that of IDO1 for D,L‐5MT and is two orders of magnitude higher than that of TDO for D,L‐5MT [50]. Similarly, the catalytic efficiency for L‐5MoT is approximately 24% of human TDO for L‐Trp. Therefore, L‐5MT and L‐5MoT may act as substrates for IDO2.

Although the physiological function of L‐5MT remains unclear, L‐5MoT exhibits anti‐inflammatory, antifibrotic, and antitumor activities and decreased levels of this compound are associated with conditions such as rheumatoid arthritis, fibrosis, and tumor progression [51, 52, 53]. Since IDO2 expression is elevated in tissues related to these pathologies, such as the synovium, colon, kidney, and liver, it is reasonable to hypothesize that increased IDO2 activity contributes to these diseases by depleting beneficial Trp derivatives, such as L‐5MoT. However, it is not yet known whether these compounds are metabolized by IDO2 in physiological settings, and further biochemical and cellular studies will be needed to evaluate their potential *in vivo* roles.

Our crystal structures highlight that when compounds that can be metabolized by IDO2 bind the catalytic site, R248 repositions to interact with the substrate. Several studies have identified a naturally occurring R248W variant, and the mutant IDO2 enzyme exhibits markedly reduced catalytic efficiency [22, 30], consistent with the notion that substituting arginine with a bulky hydrophobic tryptophan disrupts substrate positioning. At present, however, there are no studies demonstrating a robust association between R248W and specific human diseases.

Our study also revealed that IDO2 can bind to Trp derivatives in various nonproductive forms. For example, the crystal structures showed that 5HT and 5HTP assume the Conf A′ binding mode through the interaction with H143, resulting in misalignment of its indole C2 atom relative to the heme‐bound oxidant. This structural feature can explain why 5HT is not metabolized despite the observation that it binds to IDO2 with a higher affinity than to IDO1. Moreover, the N atom of the indole ring in Conf A′ cannot form a hydrogen bond with a nearby water molecule, which likely plays an important role in the enzymatic reaction. The structural change of the JK‐loop is also important for the efficient chemical reaction because it can stabilize the substrate at the catalytic site. 5HT, which lacks a carboxyl group, cannot form a salt bridge with R248 and therefore does not induce the rearrangement of the JK‐loop. In the case of 5HTP, rearrangement of the JK‐loop is incomplete because the hydroxyl group of 5HTP is positioned to form a hydrogen bond with H143, and this conformation appears incompatible with simultaneously interacting with the JK‐loop. Consequently, the JK‐loop interaction destabilizes the ligand rather than anchoring it as indicated by the weak electron density of 5HTP and the JK‐loop.

We showed that although D‐Trp binds to IDO2 with a higher affinity than to IDO1, it adopts a completely distinct binding configuration (Conf C) away from the catalytic center. This finding indicates that IDO2 does not tolerate mirror‐image substrates in the canonical binding site but rather accommodates them in alternate, nonproductive poses.

In conclusion, our study shows that IDO2 is not simply a low‐activity isoform of IDO1, but that it may function in different physiological and pathological contexts through the selective metabolism of Trp derivatives. Moreover, by revealing that IDO2 accommodates L‐Trp in a binding mode distinct from that of IDO1 despite the overall similarity of their active sites, our findings provide a structural framework that could guide the future design of IDO1‐ and IDO2‐specific inhibitors.

## Materials and methods

### Plasmid construction and mutagenesis of IDO2

The human IDO2 gene fragment encoding residues 14–420 was chemically synthesized (Eurofins Genomics K.K., Tokyo, Japan) and cloned into the pET28a(+) vector via the NcoI and XhoI restriction sites. This design, in which the first 13 amino acids of IDO2 are removed, was adopted to improve solubility and expression, as previously described [12]. The resulting construct encodes an N‐terminally truncated form of IDO2 (Δ13), fused to a C‐terminal 6xHis tag to facilitate purification.

The resulting construct was transformed into *Escherichia coli* strains XL1‐Blue MRF and BL21‐GOLD(DE3) for plasmid propagation and protein expression, respectively. Glycerol stocks of the transformed strains were prepared and stored at −80 °C.

Site‐directed mutagenesis was performed using the QuikChange Lightning Kit (Agilent Technologies, CA, USA) to generate the H143Y variant, substituting histidine at position 143 with tyrosine. The mutation was introduced into the pET28a(+)‐IDO2 WT plasmid using sequence‐specific primers, followed by DpnI digestion of the template DNA. The mutated plasmid was transformed into *E*. *coli* XL1‐Blue MRF and BL21‐GOLD(DE3), and transformants were selected on LB agar plates containing kanamycin. Verified colonies were expanded and stored as glycerol stocks.

### Expression and purification of recombinant IDO2

For protein expression, a preculture was initiated by inoculating 80 mL of LB medium supplemented with 0.1 mg·mL^−1^ kanamycin using glycerol stocks, followed by incubation at 37 °C with shaking for 12–16 h. This culture was used to inoculate 2 L of LB medium containing 0.1 mg·mL^−1^ kanamycin. The cells were cultivated at 37 °C, 140 rpm, until the OD_600_ reached approximately 0.8. Protein expression was induced by the addition of 1 mL of 200 mm isopropyl β‐d‐1‐thiogalactopyranoside (IPTG) and 2 mL of 7 mm hemin, followed by further incubation at 20 °C, 70 rpm for 18 h. Cells were harvested by centrifugation (4000 ***g***, 10 min, 4 °C), frozen at −80 °C, and stored until purification.

Cell pellets were thawed at room temperature and resuspended in an appropriate volume of lysis buffer (20 mm Tris/HCl, pH 7.8, 150 mm NaCl, 10 mm imidazole). Cell disruption was performed using ultrasonication (output: 7, duty cycle: 20%, 1.5 min × 8). The lysate was clarified by centrifugation (20 000 ***g***, 30 min, 4 °C), and the supernatant was applied to an Ni‐NTA agarose column (FUJIFILM Wako, Osaka, Japan). After washing with buffer containing 30 mm imidazole, IDO2 was eluted with 250 mm imidazole in the same buffer. The eluate was supplemented with glycerol to a final concentration of 20–30%, concentrated using an Amicon Ultra‐15 centrifugal filter device (MWCO 30000 Da) (Merk, Darmstadt, Germany), and buffer‐exchanged into 20 mm potassium phosphate buffer (KPB, pH 7.4) containing 60 mm KCl and 10% glycerol. The purified IDO2 protein was flash‐frozen in liquid nitrogen and stored at −80 °C.

### Expression and purification of recombinant IDO1

IDO1 WT was expressed and purified by the same method as described elsewhere in detail [54]. Briefly, the expression vector (pET3a) encoding the human IDO1 gene was used for transformation of *E*. *coli* BL21‐GOLD(DE3). After preculture, *E. coli* cells were cultured in the LB medium with 0.1 mg·mL^−1^ ampicillin (110 rpm, 37 °C). When the culture attained an OD_600_ of 0.6, IPTG and hemin were added to a final concentration of 5 and 7 μm, respectively, to induce protein expression, and culturing was continued (10 h, 4 °C). Cells were harvested by centrifugation (4500 ***g***) and frozen at −80 °C until use. The cell pellets were thawed and lysed in 20 mm KPB (pH 6.5). After sonication, the soluble fraction was isolated by ultracentrifugation (20 000 ***g***), and then, the supernatant was applied to a hydroxyapatite gel column (BIO‐RAD, Hercules, CA, USA). After concentration of the eluted sample containing IDO1, the solution was further purified by a CM Sepharose column (GE Healthcare, Chicago, IL, USA). The purified IDO1 protein was flash‐frozen in liquid nitrogen and stored at −80 °C.

### Plasmid construction of MBP‐IDO2

The gene encoding human IDO2 (residues 11–420) was amplified by PCR using primers and cloned into the NcoI and NdeI sites of the pET‐28a vector (Novagen, Madison, WI, USA). To improve solubility and stability, the gene encoding MBP was amplified from a pMAL‐c5X Vector (New England Biolabs, Ipswich, MA, USA) and cloned into the N‐terminus of IDO2 of pET28a(+) by using the In‐Fusion HD Cloning Kit (Takara Bio, Kusatsu, Shiga, Japan). The obtained plasmid was amplified in *E*. *coli* DH5α cells (Takara Bio). After amplification, the plasmids were purified using NucleoSpin Plasmid EasyPure (Takara Bio). The sequence of the obtained plasmid was checked by DNA sequence (Fasmac Co., Ltd., Atsugi, Kanagawa, Japan). The complete amino acid sequence is shown below, in which the Tobacco Etch Virus protease recognition sequence is written by the italic type and the IDO2 region is underlined.

#### >MBP‐IDO2

MGSSHHHHHH*ENLYFQG*MKIEEGKLVIWINGDKGYNGLAEVGKKFEKDTGIKVTVEHPDKLEEKFPQVAATGDGPDIIFWAHDRFGGYAQSGLLAEITPDKAFQDKLYPFTWDAVRYNGKLIAYPIAVEALSLIYNKDLLPNPPKTWEEIPALDKELKAKGKSALMFNLQEPYFTWPLIAADGGYAFKYENGKYDIKDVGVDNAGAKAGLTFLVDLIKNKHMNADTDYSIAEAAFNKGETAMTINGPWAWSNIDTSKVNYGVTVLPTFKGQPSKPFVGVLSAGINAASPNKELAKEFLENYLLTDEGLEAVNKDKPLGAVALKSYEEELVKDPRIAATMENAQKGEIMPNIPQMSAFWYAVRTAVINAASGRQTVDEALKDAQTTAVPLSLESYHISEEYGFLLPDSLKELPDHYRPWMEIANKLPQLIDAHQLQAHVDKMPLLSCQFLKGHREQRLAHLVLSFLTMGYVWQEGEAQPAEVLPRNLALPFVEVSRNLGLPPILVHSDLVLTNWTKKDPDGFLEIGNLETIISFPGGESLHGFILVTALVEKEAVPGIKALVQATNAILQPNQEALLQALQRLRLSIQDITKTLGQMHDYVDPDIFYAGIRIFLSGWKDNPAMPAGLMYEGVSQEPLKYSGGSAAQSTVLHAFDEFLGIRHSKESGDFLYRMRDYNQCVQALAELRSYHITMVTKYLITAAAKAKHGKPNHLPGPPQALKDRGTGGTAVMSFLKSVRDKTLESILHPRG.

### Expression and purification of MBP‐IDO2

*Escherichia coli* BL21(DE3) cells (Takara Bio) harboring the pET28a plasmid were grown in TB medium (Merck [Sigma‐Aldrich], Darmstadt, Germany) with 50 μg·mL^−1^ kanamycin sulfate (FUJIFILM Wako Pure Chemical Corporation, Osaka, Japan) at 37 °C to an OD_600_ of 0.8, and protein expression was induced by adding 0.1 mm IPTG (Nacalai Tesque, Kyoto, Japan) and 15 μm hemin (Tokyo Chemical Industry, Chuo‐ku, Tokyo, Japan), followed by incubation at 18 °C for 20 h. Cells were harvested by centrifugation at 6000 ***g*** for 30 min and resuspended in lysis buffer (20 mm Tris/HCl pH 7.5, 200 mm NaCl, 10% glycerol, 5 mm imidazole). The cells were lysed by sonication on ice, and the lysate was clarified by centrifugation at 20 000 ***g*** for 1 h. The supernatant was applied to a HiTrap TALON column (Cytiva, Shinjuku, Tokyo, Japan) pre‐equilibrated with buffer A (same as lysis buffer). The column was washed with buffer A and the bound protein was eluted with buffer B (20 mm Tris/HCl pH 7.5, 200 mm NaCl, 10% glycerol, 200 mm imidazole). Eluted fractions were pooled and concentrated using an Amicon Ultra 30 kDa centrifugal filter (Merck). During purification trials, removal of the His tag by protease treatment was inefficient. Accordingly, all subsequent experiments were performed using the His‐tagged protein. The protein was further purified by size‐exclusion chromatography using a HiLoad 16/60 Superdex 200 pg column (Cytiva) equilibrated with buffer C (20 mm Tris/HCl pH 7.5, 100 mm NaCl). The peak fractions corresponding to the monomeric form were pooled and concentrated. Purified protein was analyzed by SDS/PAGE, which showed a single band at the expected molecular weight (85 kDa). The purified protein was concentrated by an Amicon Ultra centrifugal filter, flash‐frozen in liquid nitrogen, and stored at −80 °C until use.

### Quantification of IDO concentration by pyridine Hemochrome assay

The heme content of IDOs, assumed to be in a 1:1 stoichiometric ratio with the protein, was determined using the pyridine hemochrome assay. Purified IDOs were diluted in 20 mm KPB (pH 6.5) to a final volume of 936 μL. Then, 240 μL of pyridine and 24 μL of 5 m NaOH were added to the sample. After complete reduction with an excess of sodium hydrosulfite, the UV–Vis absorption spectrum was measured using a UV–Vis spectrophotometer (UV‐2450; Shimadzu, Kyoto, Japan). The concentration of IDO was calculated based on the absorbance at 556 nm using a molar extinction coefficient of *ε*_556_ = 34.4 mm ^−1^·cm^−1^.

### UV–vis spectral analysis of IDOs

To evaluate the redox state and structural integrity of the heme moiety in purified IDOs, UV–Vis absorption spectra were recorded under three different conditions. The purified IDO samples were diluted with 20 mm KPB (pH 6.5) to a final volume of 1000 μL. The ferric (Fe^3+^) spectrum was first recorded. The sample was then reduced by the addition of an excess amount of sodium hydrosulfite, and the resulting ferrous (Fe^2+^) spectrum was measured. Subsequently, CO gas was gently bubbled through the reduced sample for 1 min, and the ferrous–CO complex spectrum was recorded. All spectra were acquired using a UV–Vis spectrophotometer (UV‐2450; Shimadzu) with a quartz cuvette at room temperature.

### Enzyme activity assay

The catalytic activity of IDO1 and IDO2 was measured by quantifying the formation of NFK, a direct oxidative product of L‐Trp, based on its UV absorbance. The assay mixture (total volume 2000 μL) contained 20 mm KPB (pH 7.4), 0.25 mg·mL^−1^ catalase, 50 μm methylene blue, 10 mm ascorbic acid, and L‐Trp at various concentrations. The reaction was initiated by the addition of IDO protein solution to achieve a final enzyme concentration of 1 μm.

Absorbance at 321 nm, corresponding to NFK, was monitored in real time at 0.1‐s intervals using a spectrophotometer (UV‐2450; Shimadzu). All measurements were conducted at 25 °C in a quartz cuvette with a 1 cm path length.

Initial reaction rate was determined from the linear portion of the absorbance‐time curve and converted to the NFK concentration using a molar extinction coefficient of 3750 M^−1^·cm^−1^. Michaelis–Menten kinetic parameters (*K*_m_ and *k*_cat_) were obtained by plotting substrate concentration versus initial rate and fitting the data to the Michaelis–Menten equation. The intrinsic clearance (*CL*_int_) was calculated as *k*_cat_/*K*_m_. All measurements were performed in triplicate (*n* = 3), and the mean was calculated. For measurements involving IDO1, the reaction buffer was adjusted to pH 6.5 using 20 mm KPB, in accordance with the enzyme's optimal activity range.

### Absorption titration

Purified IDO was diluted to a final concentration of 6.28 μm with 1× KPB (pH 6.5) and 1 m KCN was added to a final concentration of 10 mm. Experiments were performed at 25 °C. A concentrated stock solution of each compound was gradually added to the IDO solutions, and the absorption spectrum at each compound concentration was monitored by spectrophotometer (UV‐2450; Shimadzu). *K*_d_ values were calculated referring to the previous report [54]. If the differential spectrum has both a maximum point and a minimum point, the difference is plotted on the vertical axis. If one is not seen, the difference from the isosbestic point is plotted on the vertical axis, and the concentration of the compound is plotted on the horizontal axis. When *K*_d_ of the compound was sufficiently large with respect to the IDO concentration, *K*_d_ was calculated by fitting it to a hyperbolic function passing through the origin. For compounds with *K*_d_ value less than the IDO concentration, such as 1MT, the following formula was used for a similar plot:

$$\Delta A=\Delta A_{\max}\cdot \frac{{\left[\mathrm{IDO}\right]}_{\mathrm{tot}}+K_{d}+{\left[L\right]}_{\mathrm{tot}}-\sqrt{{\left({\left[\mathrm{IDO}\right]}_{\mathrm{tot}}+{\left[L\right]}_{\mathrm{tot}}+K_{d}\right)}^{2}-4{\left[\mathrm{IDO}\right]}_{\mathrm{tot}}{\left[L\right]}_{\mathrm{tot}}}}{2{\left[\mathrm{IDO}\right]}_{\mathrm{tot}}}$$

All measurements were performed in triplicate (*n* = 3), and the mean was calculated.

### Crystallization

IDO2 WT and IDO2 H143Y mutant crystals (resting state) were prepared by a micro‐seeding method. First, crystals were obtained at 4 °C by the sitting‐drop vapor‐diffusion method with a 1:1 mixture of the purified protein solution (15 mg·mL^−1^) and a reservoir solution containing 0.1 m sodium citrate tribasic dihydrate (pH 5.5) (Hampton Research, Aliso Viejo, CA, USA) and 16–20% (w/v) PEG3350 (Hampton Research). Crystallization drops were prepared on Violamo protein crystallization plates (AS ONE, Osaka, Japan) by mosquito LCP (SPT Labtech, CA, USA). Obtained crystals were crumbled by a needle and suspended in the reservoir solution to make a seed solution. Crystals for X‐ray experiments were grown at 4 °C by the hanging‐drop vapor‐diffusion method with a 1:1:0.1 mixture of the purified protein solution (15 mg·mL^−1^), the reservoir solution, and the seed solution. For the setup of the hanging‐drop vapor‐diffusion method, siliconized cover glass plates were put on a 0.5 mL sample cups (Sanplatec, Osaka, Japan) containing 400 μL reservoir solution. Before crystals were frozen in liquid nitrogen, they were soaked into the crystallization solution supplemented with 16% v/v ethylene glycol (Hampton Research) for cryo‐protection. For complex formation, crystals were soaked in cryoprotectant solution supplemented with 100 mm potassium cyanide and ligand molecules (30 mm for L‐Trp, 5HT, 5HTP, D‐Trp, D, L‐5MoT, and 15 mm for L‐5MT). Crystals were harvested with LithoLoops (Protein Wave Corporation, Nara, Japan) attached to CrystalCaps (Hampton Research) and stored in Universal V1‐Pucks (MiTeGen, Ithaca, NY, USA).

### X‐ray data collection and structure determination

All X‐ray diffraction experimental data were collected on the BL44XU beamline of SPring‐8, Hyogo, Japan. Diffraction images were obtained at 100 K using an EIGER X 16 M detector (Dectris, Philadelphia, PA, USA). The datasets of crystals obtained were collected at a wavelength of 0.9 Å. The datasets collected were processed by XDS [55] and scaled by Aimless [56]. Phase determination of the data for the IDO2 WT in the resting state was performed with Molrep [57] by using IDO1 (PDB code ID: 2D0T) and MBP (PDB code ID: 3HPI) structures separately as search models. Phases for other data were determined with Molrep by using the IDO2 resting state structure as the search model. Structural models were manually modified by Coot [58] and structural refinement was performed by Refmac5 [59] in the ccp4 suite [60] and phenix.refine in Phenix [61]. The figures in the paper were prepared by PyMOL (The PyMOL Molecular Graphics System, Version 2.0 Schrödinger, LLC.). The stereochemical quality of the final model was checked by MolProbity [62]. Data collection and refinement statistics are summarized in Tables 2 and 6.

## Conflict of interest

The authors declare no conflict of interest.

## Author contributions

SN, AT, YF, and HT designed the experiments; SN, AT, SM, NA, TF, YF, and HT performed the experiments; SN, AT, SM, NA, TF, YF, TY, TI, and HT analyzed the data; and SN, AT, YF, and HT wrote the manuscript with input from other authors.

## Supporting information


**Fig. S1.** Differential spectra of IDO1 WT titrated with D‐Trp.
**Fig. S2.** UV–Vis absorption spectra of MBP‐IDO2.
**Fig. S3.** Comparison of the catalytic site between IDO2 and human TDO.
**Fig. S4.** Superimposition of IDO structures in complex with CN^−^ and L‐Trp on TDO in complex with a dioxygen ligand and L‐Trp.
**Fig. S5.** Structural analysis of IDO2 in complex with 5HTP.
**Fig. S6.** Model structure of D‐Trp adopting Conf B in IDO2.
**Fig. S7.** Structural analysis of the IDO2 H143Y mutant in complex with 5MoT or 5MT.
**Fig. S8.** Possible van der Waals contacts between IDO2 and ligands.

## Data Availability

The raw X‐ray diffraction data collected in this study are available at the Xtal Raw Data Archive (https://xrda.pdbj.org) under the IDs corresponding to the Protein Data Bank depositions. The coordinate files and the structure factor files are deposited in the Protein Data Bank (PDB IDs: 9YZ0 for the IDO2 structure in the resting state, 9UYY for the L‐Trp complex of IDO2 WT, 9UYZ for the 5‐HT complex of IDO2 WT, 9UZ1 for the L‐Trp complex of IDO2 H143Y, 9UZ2 for the D‐Trp complex of IDO2 WT, 9UZ4 for the L‐5MT complex of IDO2 WT, 9UZ3 for the L‐5MoT complex of IDO2 WT, 21OK for the L‐5MoT complex of IDO2 H143Y, 21OM for the 5HTP complex of IDO2 WT, and 21OO for the L‐5MT complex of IDO2 H143Y).
