# Supplementary material for: Human IDO2 exhibits unique binding affinities distinct to those of human IDO1
Source: FEBS J. 2026 Mar 10;293(15):4533–53. doi: 10.1111/febs.70476 (PMC13440583; doi:10.1111/febs.70476)
Supplement: Supplementary file 1 [file FEBS-293-4533-s001.docx]

**Supporting Information for**

**Human IDO2 exhibits unique binding affinities**

**different from those of Human IDO1**

**Authors:** Shunsuke Nogi, Ayumu Takahashi, So Murakami, Nami Adachi, Tina Fujimoto, Yohta Fukuda, Taku Yamashita, Tsuyoshi Inoue, Hirofumi Tsujino

**Supplementary Figures (pp. 1-7)**

Fig. S1. Differential spectra of IDO1 WT titrated with D-Trp.

Fig. S2. UV-Vis absorption spectra of MBP-IDO2.

Fig. S3. Comparison of the catalytic site between IDO2 and human TDO.

Fig. S4. Superimposition of IDO structures in complex with CN^-^ and L-Trp on TDO in complex with a dioxygen ligand and L-Trp.

Fig. S5. Structural analysis of IDO2 in complex with 5HTP.

Fig. S6. Model structure of D-Trp adopting Conf B in IDO2.

Fig. S7. Structural analysis of the IDO2 H143Y mutant in complex with 5MoT or 5MT.

Fig. S8. Possible van der Waals contacts between IDO2 and ligands.

**Supplementary Figures**

**Fig. S1 | Differential spectra of IDO1 WT titrated with D-Trp.**

**Fig. S2 | UV-Vis absorption spectra of MBP-IDO2.** UV-vis absorption spectra in the ferric (thick line), ferrous (thin line), and ferrous-CO (dashed line) forms of MBP-IDO2.

**Fig. S3 | Comparison of the catalytic site between IDO2 (green) and human TDO (blue; PDB code ID: 5TIA).** L-Trp bound to human TDO assumes Conf A as is observed in IDO1. This structural figure was generated by PyMOL.

**Fig. S4 | Superimposition of IDO structures in complex with CN^-^ and L-Trp on TDO in complex with a dioxygen ligand and L-Trp.** IDO2: green, IDO1: purple, TDO: yellow. The dioxygen ligand as well as CN^-^ is far from the C2-C3 bond of L-Trp in Conf B. This structural figure was generated by PyMOL.

**Fig. S5 | Structural analysis of IDO2 in complex with 5HTP.** (**A**) The crystal structure of the IDO2–5HTP complex. The 2*mF*_o_-*DF*_c_ map around 5HTP are contoured at 1.0σ. (**B**) Structural comparison of the IDO2–5HTP complex with the IDO2–L-Trp complex. The inset shows difference in binding modes for L-Trp in Conf A observed in IDO1 (magenta), 5HT in Conf A’ in IDO2 (orange), and 5HTP (yellow green). The brown dashed line indicates disorder of the JK-loop in the IDO2-5HTP complex. Water molecules are illustrated by small spheres. Hydrogen bonds are shown by dotted lines with distances in Å. (**C**) Electron density (2*mF*_o_-*DF*_c_ at 1σ) of the JK-loop in the IDO2–L-Trp complex. (**D**) Electron density (2*mF*_o_-*DF*_c_ at 1σ) of the JK-loop in the IDO2–5HTP complex. These structural figures were generated by PyMOL.

**Fig S6 | Model structure of D-Trp adopting Conf B in IDO2.** D-Trp was modeled in the electron density originally assigned to L-Trp in IDO2. The resulting model revealed that the distance between the hydroxyl group of T385 and the α-carbon atom of D-Trp is shorter than the sum of their van der Waals radii (2.6 Å). This steric hindrance indicates that D-Trp cannot be located at the catalytic site of IDO2. This structural figure was generated by PyMOL.

**Fig. S7 | Structural analysis of the IDO2 H143Y mutant in complex with 5MoT or 5MT.** (**A**) The crystal structure of the IDO2–5MoT complex. (**B**) Structural comparison of the IDO2–5MoT complex with the IDO2–L-Trp complex. (**C**) The crystal structure of the IDO2–5MT complex. T (**D**) Structural comparison of the IDO2–5MT complex with the IDO2–L-Trp complex. The sigma-A-weighted 2*F*_o_-*F*_c_ map around ligands are contoured at 1.0σ. Water molecules are illustrated by small spheres. Hydrogen bonds are shown by dotted lines with distances represented in Å. (**E**) Structural comparison of the IDO2–5MoT complex with the IDO2 H143Y–5MoT complex. (**F**). Structural comparison of the IDO2–5MT complex with the IDO2 H143Y–5MT complex. These structural figures were generated by PyMOL.

**Fig. S8 | Possible van der Waals contacts between IDO2 and ligands.** (**A**) Crystal structure of the IDO2–5MT complex. (**B**) Crystal structure of the IDO2–5MoT complex. (**C**) Comparison between the IDO2–5MT complex and the IDO1-L-Trp complex (representative of a Conf A ligand). (**D**) Comparison between the IDO2–5MoT complex and the IDO1-L-Trp complex. In (**C**) and (**D**), possible van der Waals interactions between ligands and L146 of IDO2 are shown by dotted lines with distances represented in Å. (**E**) Distances between 5MT and C129 in IDO1. (**F**) Distances between 5MoT and C129 in IDO1. Contacting atoms are shown as spheres. These structural figures were generated by PyMOL.
